# Spatial and Temporal Organization of Chromosome Duplication and Segregation in the Cyanobacterium *Synechococcus elongatus PCC 7942*

**DOI:** 10.1371/journal.pone.0047837

**Published:** 2012-10-24

**Authors:** Anna H. Chen, Bruno Afonso, Pamela A. Silver, David F. Savage

**Affiliations:** 1 Department of Systems Biology, Harvard Medical School, Boston, Massachusetts, United States of America; 2 Wyss Institute for Biologically Inspired Engineering, Harvard University, Boston, Massachusetts, United States of America; 3 Department of Molecular and Cell Biology and Department of Chemistry, University of California, Berkeley, California, United States of America; Center for Genomic Regulation, Spain

## Abstract

The spatial and temporal control of chromosome duplication and segregation is crucial for proper cell division. While this process is well studied in eukaryotic and some prokaryotic organisms, relatively little is known about it in prokaryotic polyploids such as *Synechococcus elongatus PCC 7942,* which is known to possess one to eight copies of its single chromosome. Using a fluorescent repressor-operator system, *S. elongatus* chromosomes and chromosome replication forks were tagged and visualized. We found that chromosomal duplication is asynchronous and that the total number of chromosomes is correlated with cell length. Thus, replication is independent of cell cycle and coupled to cell growth. Replication events occur in a spatially random fashion. However, once assembled, replisomes move in a constrained manner. On the other hand, we found that segregation displays a striking spatial organization in some cells. Chromosomes transiently align along the major axis of the cell and timing of alignment was correlated to cell division. This mechanism likely contributes to the non-random segregation of chromosome copies to daughter cells.

## Introduction

**Figure 1:**
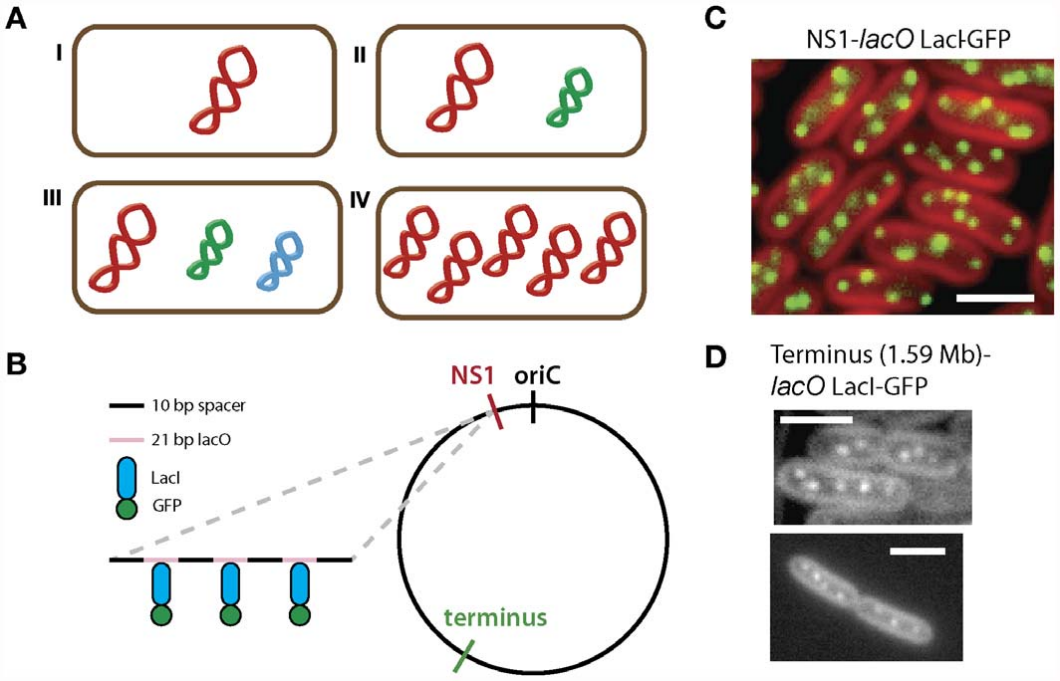
Chromosomes in the polyploid bacterium *S. elongatus* can be visualized using a fluorescent repressor-operator system. (A) Bacteria contain different genomic arrangements. Here, each color represents a different chromosome. They can possess a single copy of one chromosome (I), or have multipartite genomes (II-III) with one large chromosome (red) and one or more smaller chromosomes (green and blue). Some species of bacteria, such as cyanobacteria *Synechococcus elongatus* PCC 7942, are polyploid. That is, they have multiple complete copies of one chromosome (IV). (B) Chromosomes can be tagged and observed *in vivo* using a fluorescent repressor-operator system. *lacO* arrays were integrated either near the origin of replication (NS1) or the predicted terminus in the *S. elongatus* chromosome. 10 bp spacers with random sequences were inserted between the operator sites to avoid recombination (black). The protein fusion Lacl-GFP (blue and green) bound to multiple repeats of its cognate *lacO* operator site (pink). (C) The fluorescent repressor-operator system from (B) was transformed into *S. elongatus.* The origins of replication of each chromosome appear as foci (green) in cells (red) when imaged using wide field fluorescence microscopy. Origins of replication are seen throughout the cell. (D) Cells with *lacO* arrays integrated near the putative terminus region at 1.59 Mb in the genome were visualized. Foci appear throughout the cell, similar to (C).

Genomic DNA replication and segregation are fundamental processes crucial to survival for all organisms. This process has been well studied in many bacterial species, including *Escherichia coli* [1], *Bacillus subtilis* [2]–[4], and *Caulobacter crescentus* [5], [6]. Most of these organisms possess a single copy of one, two or three different chromosomes (Fig. 1A, I-III). In contrast, the cyanobacterium *Synechococcus elongatus PCC 7942* has multiple copies of its single chromosome – estimates suggest between three to six copies [7], [8] (Fig. 1A, IV). To date, little is known about the dynamics of replication and segregation in prokaryotes with multiple copies of a single chromosome.

Most studies of replication and segregation so far have been conducted in monoploid bacterial species. In many of these organisms, replication timing and synchrony is strictly regulated [9]. In *E. coli*, for example, all origins fire synchronously at a fixed cell size per origin (initiation mass) that is independent of the growth rate [5], [10]. Synchrony is tightly coupled to cell division cycles and ensures that daughter cells receive the correct number of chromosomes. However, regulating timing of replication may not be as important for proper cell division in polyploid organisms.

In addition to timing of replication, the spatial localization of replication is important for proper cell division. In *E. coli*, newly synthesized chromosomes appear in the cell center or at the quartile points along the long axis of the cell [11]. Replication forks appear at the origin of replication, separate into two sister replisomes that migrate to opposite cell halves as replication proceeds and returns to mid-cell as replication ends [11]. In *C. crescentus*, replisomes move towards the middle from the cell poles [12]. In this study, we probe the localization dynamics of chromosome replication in *S. elongatus*. We also investigate the stringency of the spatial organization of replication.

It has been suggested that organisms with multiple chromosomes do not require an active segregation mechanism, since given a large number of chromosomes, it is likely that each daughter cell will receive at least one copy [4]. This is analogous to high-copy plasmid systems, which typically lack an active segregation mechanism [13].

In order to better understand chromosome replication and segregation in the polyploid organism, *S. elongatus,* we tagged and visualized the chromosome and the replisome using a fluorescent repressor-operator system. Chromosome count and localization data was collected. We probed the spatial organization of *S. elongatus* chromosome segregation and found that, contrary to previously suggested models, a surprising alignment occurs during the process. In addition, we calculated the timing of replisomes and the diffusive dynamics of replication. We found that chromosome number correlates with cell length but that chromosome duplication timing is asynchronous. Thus, while duplication is correlated to cell length, it is not coupled to cell division. Spatially, chromosome duplication occurs at random locations in the cell, but movement of each individual replisome remains confined after initiation. Together, these results elucidate chromosomal replication and segregation dynamics in a polyploid prokaryote.

## Materials and Methods

### Bacterial Strains and Growth Conditions

The wild-type *Synechococcus elongatus* PCC 7942 strain was acquired from the American Type Culture Collection (ATCC). *S. elongatus* cells were grown in solid BG11 medium following standard protocols with an illumination of 2000 lux at 30°C [14]. *S. elongatus* were transformed following standard protocols by incubating cells overnight in the dark with 100 ng of plasmid DNA and plating on selective media [15]. Antibiotics (kanamycin, spectinomycin, or chloramphenicol) were used at a concentration of 5 µg/ml. To prevent disruption of chromosome replication during growth, 200 µM isopropyl β-D-1-thiogalactopyranoside (IPTG) was added to the media. Cells were then replica plated onto media with 50 µM IPTG [19] for visualization and further experiments.

### Plasmid Construction

All cloning, unless otherwise stated, was done using a Biobrick-like strategy (SpeI as the upstream site and XbaI-HindIII-NotI as the downstream sites) [16]. 21 bp *lacO* operator sites were assembled with random ten bp spacers. *lacO* arrays were obtained from pLAU443 [17]. Two *lacO* arrays, with 120 *lacO* sites each, were then assembled with a kanamycin resistance marker inserted between them. Using Nhe1 and SalI restriction enzymes, this series of *lacO* arrays was then cloned into the neutral site 1 vector pAM2314 [18] or a vector containing homology regions to the terminus at 1.59 Mb in the *S. elongatus* chromosome. In the same vector LacI, fused to either the superfolder variant of green fluorescent protein (GFP) or yellow fluorescent protein (YFP) was inserted.

### Image Acquisition and Analysis

Cells were plated onto BG11+50 µM IPTG [19] +2% agarose pads, which were transferred to a glass bottom dish (MatTek) for imaging. A Nikon TE-2000 microscope with a 100×1.4 numerical aperture objective equipped with an ORCA-ER CCD camera was used. Image acquisition utilized custom software, written using MATLAB (Mathworks), which interfaced with the microscope control package µManager [20]. Lighting necessary for cell growth during time lapse microscopy was controlled via a network AC power controller (IP Power 9258T), which also interfaced with MATLAB. Image analysis was performed using ImageJ [21], custom software written in MATLAB using the Image Processing Toolbox, and MicrobesTracker [22]. Cells were segmented using phase contrast images and cell size was calculated. Chromosomes were identified as foci in fluorescent images and their location and number calculated. Tracking and segmentation were verified manually and corrected as necessary.

### Single-Stranded-Binding (SSB) Protein Visualization

SSB protein genes were cloned from *S. elongatus*, fused to mOrange, and cloned into the neutral site 3 vector using methods described above. The resulting plasmid was transformed into cyanobacteria either alone or with the LacI-*lacO* plasmid and visualized using methods described above.

### Fluorescently Labeled Nucleotides Incorporation and Imaging

Cells were grown in the presence of 0.3% pluronic F-68 to OD750 = 0.4. Pluronic F-68 concentration was then elevated to 3% and fluorescently labeled nucleotides tetramethylrhodamine-5–2′-deoxy-uridine-5′-triphosphate (Roche) were added at a final concentration of 3 µM. After growth to late log phase, cells were washed in PBS and imaged as described above.

## Results

### Fluorescent Tagging of the Genome Using a Repressor-operator System Reveals the Spatial Localization of Origins and Termini of Chromosomes *in vivo*

Organization of chromosomes has been studied in cyanobacteria using DAPI and fixed-cell staining methods [23], [24]. However, these methods only give a static and low-resolution image of chromosome localization. In order to visualize and quantify chromosome dynamics *in vivo*, we used a fluorescent repressor-operator system (Fig. 1B). This system uses fluorescently-tagged DNA-binding proteins that bind to their cognate recognition sequences. Multiple proteins bound to operator arrays then appear as foci when imaged using fluorescence microscopy [25], [26]. In our case, we used the LacI repressor fused to either yellow fluorescent protein (YFP) or the superfolder variant of the green fluorescent protein (GFP) as our DNA binding protein [27]. Simultaneously, an array of 240 *lacO* operator sites [1] was inserted using homologous recombination at various positions in the chromosome (Fig. 1B).

The precise location of the origin of replication (*oriC*) and the terminus region (*ter*) was predicted using the program Ori-Finder on the *S. elongatus* chromosome [28] and was recently confirmed with experimental data [29]. This analysis revealed that the origin of replication is located at the region defined as the start in the current chromosome sequence from NCBI. It is the intergenic region between *dnaN* and *ccbZp* and contains 11 *dnaA* boxes (consensus sequence *TTTTCCACA)* [28]. Interestingly, the *dnaA* gene, which is usually found near *oriC* in other species, was found elsewhere in the genome (1.1 Mb). The terminus region was not clearly defined via either GC skew or base disparity [28]. Due to the highly recombinant nature of the *S. elongatus* genome, the GC skew plot does not display a clear V-shape typical of organisms such as *E. coli* [30]. We reasoned that the terminus would be close to the region with the highest peak of GC disparity (Fig. S1, green line) so we inserted the *lacO* array at 1.59 Mb in the chromosome.

Visualization of tagged *oriC* showed multiple distinct foci throughout the cell (Fig. 1C). This is unlike previous observations of *oriC* localization in *E. coli* and *B. subtilis,* where *oriC* were replicated and maintained at the poles of cells [31], [32]. Tagged termini also displayed multiple foci throughout the cell (Fig. 1D), whereas previously, termini in *E. coli* were found to migrate from poles to mid-cell during cell division [33]. Also, the origin and terminus in *E. coli* are spatially separate, confined to distinct regions of the cell [1]. We did not find such spatial specificity in *Syenchococcus* (Fig. 1D, bottom). Together, these data confirm earlier studies that *S. elongatus* does indeed have multiple copies of its chromosome throughout the cell cycle [34]. They also reveal that the localization of the *oriC* and terminus in *Syenchococcus* is distinct from that in other bacteria. The strains with the array integrated near the *oriC* showed a better signal-to-noise-ratio, so this strain was used in subsequent experiments.

### Chromosome Duplication is Correlated to Cell Length and not Coupled to Cell Division

Using a custom made algorithm developed in MATLAB, we quantified the number of fluorescent foci (tagged *oriC*), representing the number of chromosome copies in each cell. The algorithm also allowed us to quantify cell boundaries and cell length using phase contrast images.

**Figure 2:**
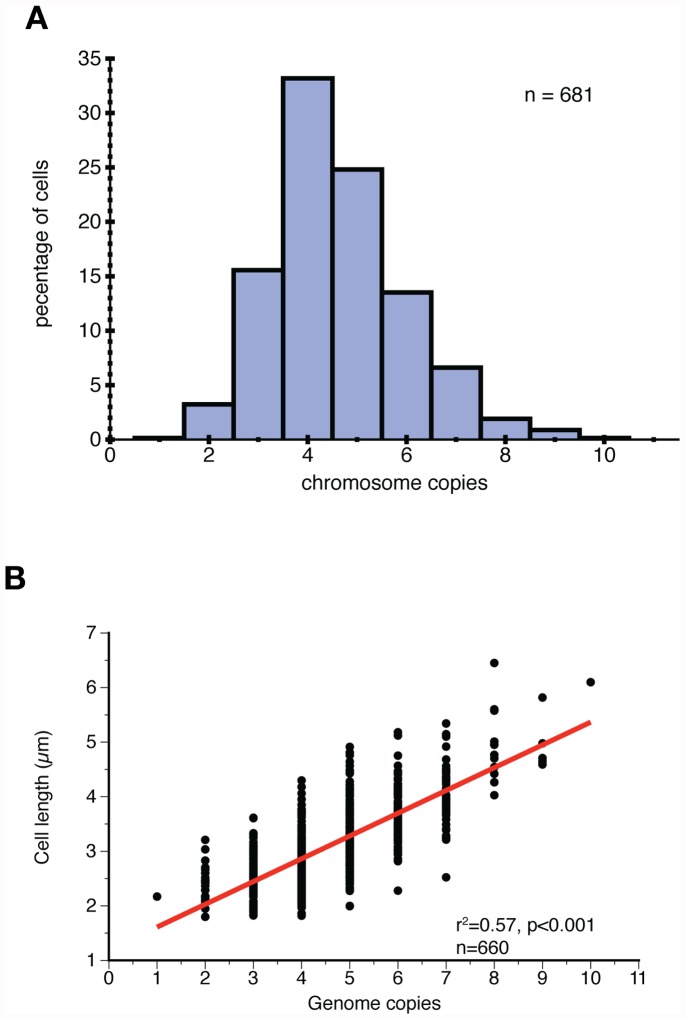
Chromosome duplication is correlated to cell length. (A) Distribution of chromosome number per cell is not significantly different from a log-normal distribution (n = 681, χ^2^ goodness of fit test, h = 0, p = 0.2621). Most cells contain 4 chromosomes with values ranging from 1 to 10. (B) Chromosome copy number is correlated to cell length (n = 660, r = 0.7519, p<0.001), suggesting chromosome duplication is coupled to cell growth.

We found that the chromosome copy number distribution was not significantly different from a log-normal distribution (n = 681, χ^2^ goodness of fit test, h = 0, p = 0.2621) with a mean of 4.62 copies per cell and a median and mode of 4 copies per cell (Fig. 2A). Cells harbored 1–10 chromosome copies. Interestingly, some cells contained an odd number of chromosomes and chromosome copy numbers other than 2^n^ copies. This observation does not fit the model typically observed in single replicon prokaryotes, where replication occurs synchronously [9]. Instead, it is similar to observations of asynchrony in *E. coli* replication mutants [35]. The chromosome copy numbers hint at asynchronous DNA replication, supporting previous fjndings of constant DNA synthesis rate over time; that is, DNA replication is not coupled to cell division [34].

We found that larger cells contained higher number of chromosome copies compared to smaller cells, strongly supporting a model where chromosomes replicate at a constant rate during growth. By observing chromosome numbers in growing cells, we found a linear correlation between cell length and number of chromosomes (n = 660, r^2^ = 0.57, p<0.001) (Fig. 2B, red line). These results suggest that chromosome duplication is correlated to cell growth.

### Replication Timing is Asynchronous: Only One Replisome is found in Most Cells at Any Given Time

In order to better understand the timing and spatial localization of single replication events *in vivo,* we fluorescently tagged single-stranded-binding (SSB) proteins. SSB proteins play a fundamental role during chromosome replication, coating single-stranded DNA that is temporarily exposed; thereby, preventing it from degradation [36]. Approximately 30 SSB proteins localize to the replisome in *E. coli*, and this method has been used extensively to track replisomes in other organisms [11], [37].

**Figure 3:**
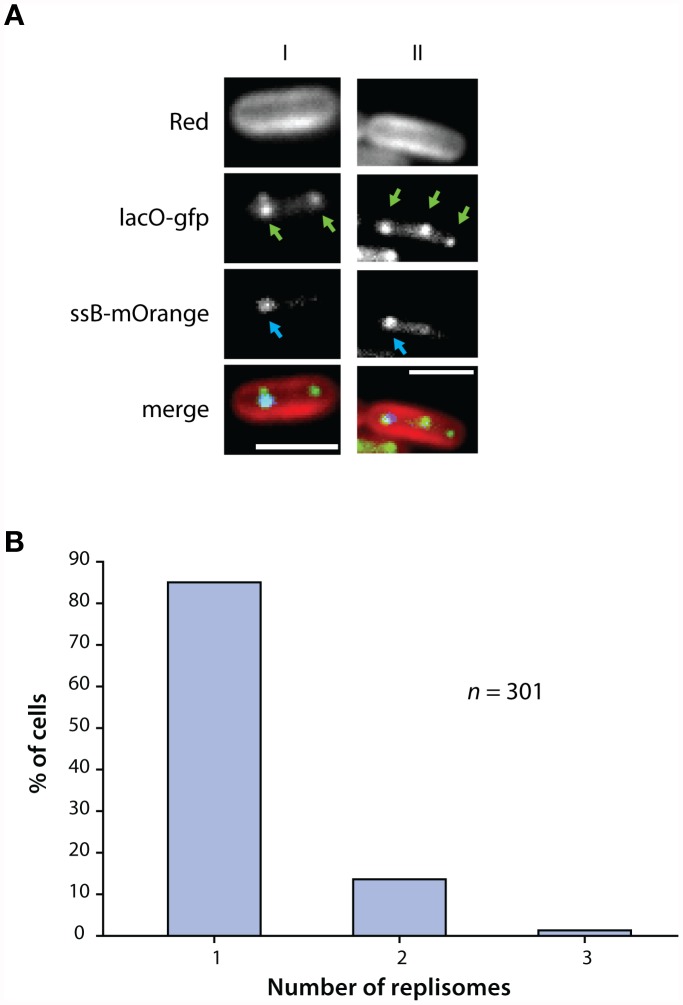
Chromosome duplication is asynchronous and is not coupled to cell division. (A) Single stranded binding (SSB) protein was tagged with mOrange. These were co-expressed in cells with LacI-GFP (NSl-*lacO*). Replisome localization appeared as foci and co-localized with tagged chromosomes (merge), indicating that tagging did not interfere with functioning of SSB. A cell with two chromosomes (left, green arrows) only contains one actively replicating chromosome (left, blue arrows). A cell with three chromosomes (right, green arrows) also only has one replisome (right, blue arrows). (B) Most cells contain one actively duplicating chromosome (85%), while the remaining contain two or three replisomes. Since most cells contain multiple chromosomes but only one replisome, this shows that chromosome duplication is asynchronous.

Cyanobacterial SSB protein was fused to mOrange and expressed in cells with the origin of replication tagged with GFP (as described above). We found that SSB foci (SSB-mOrange) were co-localized with Origin-GFP foci present in the cell (Fig. 3A), indicating that tagging did not interfere with SSB function. SSB foci, therefore, correspond to active chromosome replication occurring in the cell. We found that at any given time, 85% of cells contain just one replisome, while 13.6% have two and only 1.3% have three (Fig. 3B). Since most cells contain only one actively replicating chromosome (SSB-mOrange foci) but more than one chromosome (Origin-GFP foci), our data show that, in most cells, only one copy of the chromosome is being replicated at any given time. This, along with chromosome number data (Fig. 2A) suggests that replication occurs asynchronously in *S. elongatus*.

### Chromosome Duplication is Spatially Randomly Distributed

Since we found that cells containing multiple chromosomes typically replicate a single chromosome at a time (Fig. 3), we hypothesized that new chromosomes may be synthesized at a particular location in the cell, either at the poles or mid-cell. We investigated the spatial localization of the replication event and of newly synthesized chromosomes to determine if there is a spatial preference for replication.

**Figure 4:**
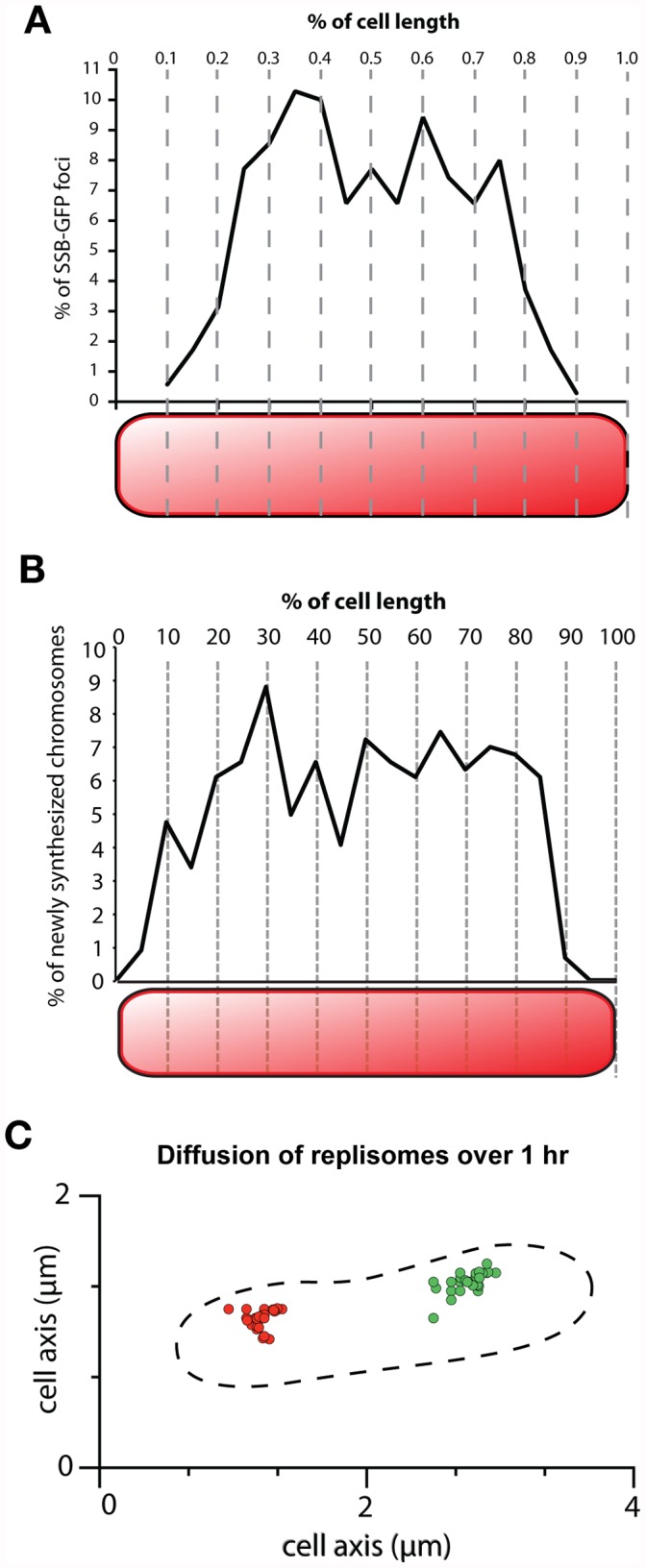
Replication occurs in a spatially random manner but replisomes undergo constrained movement. (A) Chromosome duplication events have no spatial preference within the cell. Cells (n = 297) were sub-segmented along the major axis into 20 smaller regions and SSB foci localization was binned. The distribution of SSB localization was not found to be significantly different from a uniform distribution (KS test, h = 0, p = 0.4867, k = 0.0456), suggesting spatially random replication events. Cell poles were excluded in this analysis because of boundary effects due to reduced cell volume and the volume of the nucleoid decreasing the likelihood of SSB foci found at the edges of rod shaped cells. (B) To confirm the random localization of chromosome duplication, newly synthesized chromosomes were visualized using fluorescent nucleotide incorporation. Fluorescent foci also show a uniform distribution (KS test, h = 0, p = 0.1027, k = 0.0579), confirming the results shown in (A). (C) Two replisomes (red and green) were tracked over the time scale of a complete replication cycle with images taken every two minutes. Replisomes show restricted occupation of domains in the cytoplasm, remaining close to their initial position, suggesting that chromosomes are spooled through the replisomes.

To accomplish this, we segmented cells using a custom MATLAB algorithm and sub-segmented each cell along the major axis into 20 smaller regions. SSB foci location was distributed into these bins based on their distance from the pole of the cell along the major axis (Fig. 4A). This distance was normalized to the total length of the cell. We found that the distribution of SSB localization was not significantly different from a uniform distribution (Kolmogorov-Smirnov test, h = 0, p = 0.4867, k = 0.0456), suggesting random localization of replisomes. That is, duplication is equally likely to begin at any point along the length of the cell in the inner quintiles (20%–80%). SSB at the poles of cells were not included in this analysis, since nucleoid volume results in reduced probability of the replisome appearing at the edge of the cell. In addition, SSB foci are less likely to be found at the poles due to decreased cell volume at the ends. From this data, we found a striking absence of spatial preference for beginning replication.

In order to confirm our finding that chromosome replication occurs in random locations throughout the cell, we also fluorescently labeled newly synthesized DNA. To do so, we permeabilized cells using pluronic F-68 to allow uptake of fluorescently labeled nucleotides [38]. After cells grew to late log-phase, incorporation of fluorescent nucleotides into the chromosome was seen as foci. The images were automatically segmented and binned as described earlier. There seemed to be no spatial preference in newly synthesized DNA (Fig. 4B). Chromosome localization in the inner deciles (10%–90%) of the cell’s major axis was not significantly different from a uniform distribution (KS test against uniform distribution. h = 0, p = 0.1027, k = 0.0579). We conclude that there is no spatial preference for chromosome duplication in the cell, confirming our previous results (Fig. 4a) from replisome localization data. These findings are in contrast to findings on chromosome replication in *E. coli*, which are preferentially localized to the center or the quartile points along the major axis (in cells with one and two replisomes respectively), as well as chromosome replication in other organisms, where chromosomes preferentially replicate at the poles or the middle of the cell [2], [39], [40].

### Replication Fork Movement is Constrained

We concluded that replisomes appear at random locations throughout the cell, but how does their localization change over the course of a single duplication process? To investigate dynamic behavior of replisomes, time lapse imaging of SSB foci was used to track movement of replication forks. We followed replisomes over time, tracking individual foci every 5 seconds. We calculated a diffusion coefficient of 6.24×10^−5^±4×10^−5^ µm^2^/s (*n* = 20) for replication forks, similar to the reported values for *E. coli* (∼10^−4^ µm^2^/s) [11], [41].

In order to determine whether movement is constrained over the time scale of a complete replication cycle, we tracked replisomes for one hour, acquiring images every two minutes (Fig. 4C). We found that replisomes were confined to a region of the cytoplasm, remaining close to their initial position. This behavior was observed in cells containing one or two replisomes, suggesting that this is not merely the result of observing two strands of the same chromosome. The small amount of movement over the time scale of replication suggests that replisome motion is constrained to the local cytoplasmic region in which it assembled. This is distinct from *E. coli* or *C. crescentus* in which replication follows directed motion between mid-cell and the edges of the cell. The data suggest that chromosomes are spooled through the replisomes rather than replisomes tracking along the chromosome. Together, these data give us an understanding of the spatial organization of chromosome duplication in *S. elongatus*: duplication starts in random locations throughout the cell and as duplication proceeds, the replisomes remain stationary.

### Chromosomes Transiently Align During the Cell Cycle

The lack of spatial preference of duplication events supports the current model that organisms with multiple chromosome copies do not have an active chromosome segregation system [4]. It is currently believed that, just as in a high copy number plasmid system [13], a large number of chromosomal copies abolishes the need for an active segregation system since it is highly likely that each daughter cell obtains at least one copy by random. We surmised that our GFP-lacI-*lacO* method of visualizing chromosome dynamics *in vivo* would provide further insights into the dynamics of multiple chromosomes during segregation and allow us to follow up on previous works [24]. We found striking spatial organization, contrary to earlier models of random chromosome segregation in polyploid organisms.

**Figure 5:**
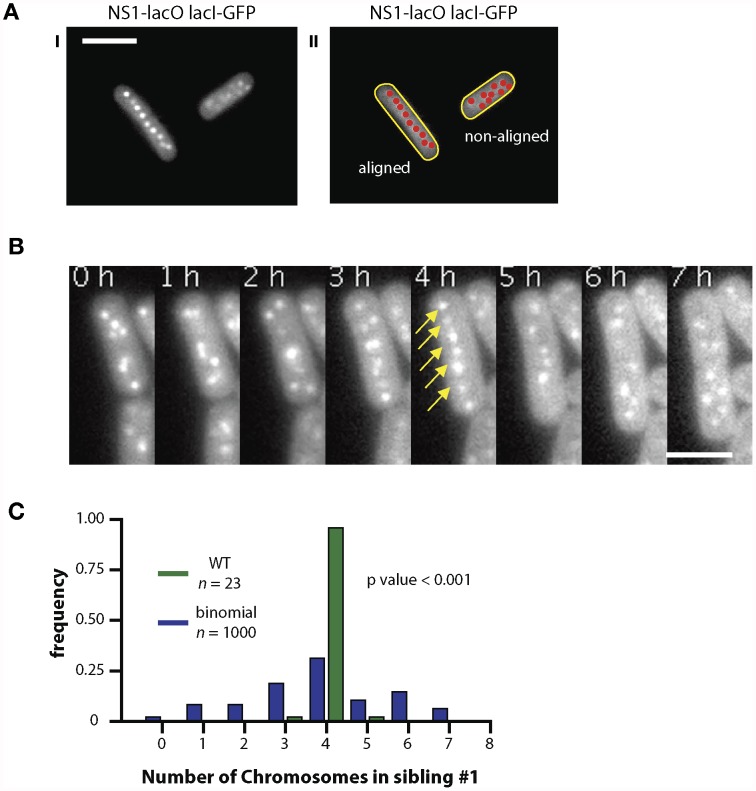
Chromosomes align transiently before non-random segregation occurs. (A) Chromosomes in cells visualized through Lacl-GFP in an NSl-*lacO* background showed two different spatial arrangements. The chromosomes are either aligned along the major axis of the cell (I) or randomly localized (II). (B) Time-lapse imaging of single cells revealed a transient alignment of the chromosomes (4 hours, yellow arrows) approximately three hours before the cell entered cytokinesis. (C) Almost all sibling cells descended from mother cells containing eight chromosomes inherit four chromosomes, thus chromosome segregation is highly non-random.

Cells in a freely growing population displayed one of two phenotypes. Most of the cells displayed randomly localized chromosomes (85%, n = 289) (Fig. 5A right). However, some cells displayed chromosomes aligned along the major axis of the cell (Fig. 5A left). This surprising behavior prompted us to analyze the spatial arrangement of the chromosome copies over time. We tracked growing cells every hour for eight hours after which we could no longer distinguish signal from background (Fig. 5B). We found that chromosomes transiently aligned during the cell cycle: collapsing towards the middle and aligning evenly spaced along the major axis of the cell (Fig. 5B, 4 hrs, yellow arrows). Shortly after (1 hr), spatial arrangement was lost. This transient spatial arrangement took place either one or two times during the acquisition window of 8 hours. In some of the cases, partial alignment was observed; that is, not all of the chromosomes aligned. This process was correlated to cell division, hinting that this process is mainly driven by the cell’s commitment to division and may help maintain high fidelity chromosome segregation.

### Chromosomes are Non-randomly Segregated

The transient alignment of chromosomes prompted us to hypothesize that chromosome segregation is not random, and that cells harboring the transient alignment have a high fidelity of chromosome segregation. This would be analogous to the spatial organization previously found regulating segregation of carboxysomes, microcompartments involved in the “carbon concentrating mechanism” in *S. elongatus* [42]. If true, chromosome segregation organization would be another example of order in bacteria, which were previously thought to be homogenous “bags of protein” with little to no internal organization [43]. To test our hypothesis, we assayed chromosome segregation by quantifying the number of chromosomes each daughter cell inherited after cytokinesis of mother cells (n = 48) that had 8 chromosome copies (Fig. 5C). We found a striking difference between the experimental results and a predicted binomial distribution based on random segregation to daughter cells (Fig. 5C Lilliefors test, P<0.001). Cells with 7, 9, and 10 chromosome copies were likewise found to undergo nonrandom segregation (Lilliefors test, P<0.001).

## Discussion

The goal of this research was to understand the dynamics of chromosome replication and segregation in *S. elongatus*. To do so, we used the GFP-lacI-*lacO* fluorescent repressor-operator system to visualize chromosomes *in vivo.* Similar analyses have been performed in other bacterial species, including *E. coli*, and *B. subtilis* [1], [44]. Here, however, we investigate live chromosomal dynamics in a bacterial species with multiple chromosomes. Having multiple copies of a single chromosome changes the parameters of the biological problem of replication and segregation that the organism must solve in order to successfully pass on its genetic information. The replication and segregation organization we found in *S. elongatus* differs from that in *E. coli* or other bacteria, and the difference can be attributed to the different challenges these bacteria face in passing on chromosomes to the next generation.

Bacterial chromosomal DNA is compacted into a nucleoid. In *E. coli*, the terminus and the origin can be overlapping or far apart from each other depending on the timing of replication and the cell cycle [45]. In our study, we labeled either the terminus or the origin of replication and analyzed origin movement in replication and segregation; however, different parts of the chromosome may be localized differently. We could not maintain the strain with the *lacO* array integrated at the terminus regions for long periods of time and we speculate that this may be due to a higher level of recombination occurring in that region of the chromosome. Integrations at other regions of the chromosome will clarify whether this is a global trend or specific to that region of the chromosome.

**Figure 6:**
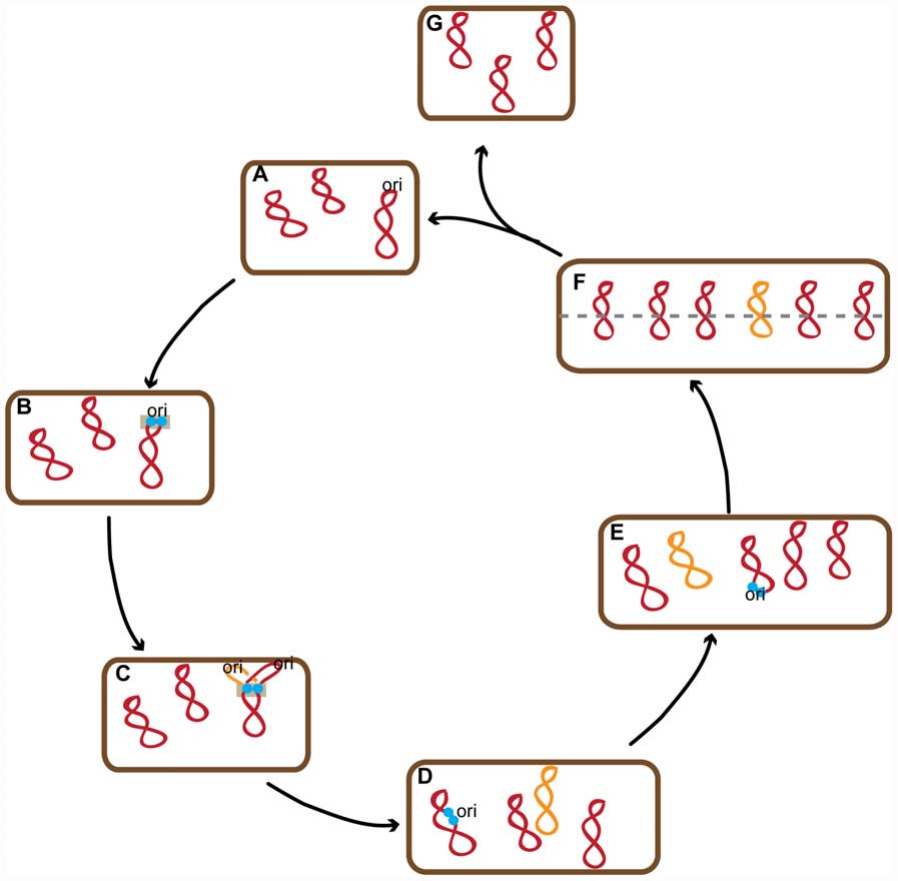
Model of chromosome replication and segregation in the polyploid bacterium *S. elongatus* PCC 7942. *S. elongatus* possess multiple copies of a single chromosome, shown in red (A). Chromosomes are duplicated asynchronously and coupled to cell growth (B, D, E). Newly synthesized chromosomes (orange) are synthesized in a spatially random manner (D,E,F). Replisomes (blue) assemble on a spatially random chromosome (B,D,E), but once initiated, their motion remains confined within the same region of the cell (grey box, B to C). Chromosomes transiently align (F) before non-random segregation and cytokinesis (F to G &A).

Our studies suggest that *S. elongatus* chromosome origins are randomly distributed throughout the cell. This is in contrast to specific localization of origins and termini in other bacterial species. In *E. coli* for example, the *oriC* is localized mid-cell and the terminus region is located at the poles of the cells [32]. In *C. crescentus*, both the *oriC* and *ter* are located at the poles [46]. In *V. cholerae*, one of its two origins is localized to the mid-cell, and the other is at the pole [47]. *B. subtilis* origins are located at the poles [31]. In these other species, replicated chromosomes initially occupy the same regions and require separation for proper segregation to occur. The relative positions of *S. elongatus* origin and terminus are still unclear. Two color experiments with tagging at both the terminus and origin (or other regions of the chromosomes) would help elucidate details of replication and segregation: how the DNA strand is situated within the cell during different phases of replication and segregation. It will also answer how compact and region-excluded each chromosome is from its neighbors. The chromosomes may occupy distinct territories as has been previously observed in DAPI stained chromosomes [24]. The existence of local regions occupied by a single nucleoid can be advantageous for an organism with multiple chromosomes as it can undergo cytokinesis without the need to untangle chromosomes using FtsK or similar proteins.

Chromosome replication and cell division are fundamentally linked in most organisms [48], that is, replication must occur exactly once before (or during) cell division. Both events are believed to be regulated by mass doubling time, i.e. how long it takes for a cell to grow to double its mass [5]. Contrary to these studies in other organisms, asynchrony of chromosome replication has been observed in *S. elongatus* [8], [29]. Indeed, recently, Watanabe et al. showed that cyanobacterial chromosomes replicate asynchronously based on mapping analysis and fixed cell staining methods [29]. They also suggested that while replication is still coupled to cell division (peaking at a few hours before cell division occurs), it is less stringently coupled than in *E. coli* or *B. subtilis*. In addition, cell division has been found to be gated by circadian rhythm [34], that is, cyanobacteria do not divide at night. Thus, timing of chromosome dynamics may be regulated by the circadian cycle. We found that replication in *S. elongatus* is independent of cell division and is instead correlated to cell length. We also found that chromosomes were replicated multiple times every cell division. Both replication and cell division may still be dependent on cell mass, but if so, the threshold of activation is much lower for chromosome replication than it is for cell division in *S. elongatus*. Observing chromosome copy number in cells where cytokinesis is inhibited may uncouple these dependencies. In addition, growing cells in multiple conditions leading to different cell division rates would also clarify the dependence between cell growth and chromosome copy number.

Several mechanisms ensure chromosomal replication takes place once every cell cycle and simultaneously from all origins in *E. coli* as well as in other prokaryotes [35], [45]. These regulatory cellular processes give rise to a chromosome copy distribution where all possible values can be written in the form 2^n^, where n is an integer. A Gaussian-like distribution of chromosome copy number similar to the one we observed has been found in *E. coli DnaA* mutants, which have disrupted initiation of replication [35], [49]. Such a distribution could arise from the inability of some chromosomes to complete a single round of replication after the cell initiates synchronous replication. Alternatively, asynchronous initiation could also result in distributions containing copy numbers other than 2^n^. We found that most cells only have one replisome and therefore only one actively replicating chromosome at a time. This supports asynchronous initiation as the mechanism that resulted in the observed Gaussian distribution of chromosome copy number.

There are two competing models for how replisomes proceed in DNA replication [37]. The first is the independent replisome model, in which replisomes and replication forks track along the stationary chromosome. The second is the spooling replisome model, in which DNA is “spooled” through relatively stationary replisomes. A variation of the spooling model is the factory model in which the left and right replisomes are physically coupled throughout DNA replication. While early results favored a spooling model, recent results have shown that sister replisomes transiently separate. Another study observed that sister replisomes appear together early in S-phase but afterwards independently track DNA until they meet again as they near the terminus region. While our results suggest the spooling model is more likely in *S. elongatus*, we cannot rule out the independent model given the resolution limits of wide-field microscopy, especially if chromosomes each occupy exclusive local cytoplasmic regions during replication.

The spatial organization of chromosome origins along the major axis of the cell was observed for short periods (<1 hr) of the cell cycle. This spatial organization of chromosomes is surprisingly similar to eukaryotic mitosis, in which chromosomes align during metaphase before migration to the poles. However, in our current study, an active mechanism maintaining the organization was missing. Most other mechanisms of prokaryotic spatial organization studied such as carboxysome localization in *S. elongatus* [42] as well as low-copy plasmid segregation in *E. coli* [50], [51], are maintained constantly throughout the cell cycle. We speculate that *S. elongatus* may not require an active segregation mechanism throughout the entire cell cycle in part because entropic forces may not be sufficiently disruptive to the arrangement of chromosomes post alignment and before cytokinesis. That is, due to having multiple chromosomes, *S. elongatus* do not have a stringent segregation problem and may not need constant organization. The transient alignment may enrich for rather than actively impose even segregation.

### Conclusions

We have shown that the multiple copies of the chromosomes in *S. elongatus* can be tagged and tracked in living cells. Our model and findings are summarized in Fig. 6. Chromosomes are replicated in a linear-like fashion correlated with cell length in growing cells. By tracking replisomes *in vivo* we show that chromosome replication takes place in a confined region of the cytoplasm in accordance with the spooling replisome model and that chromosome replication does not happen preferentially in specific locations of the cell. Finally, we show *S. elongatus* segregates chromosomes to daughter cells in a non-random fashion, which we speculate may be the result of a cellular process that transiently organizes the chromosomes just before completion of cell division.

While this manuscript was in preparation, Jain et. al independently came to similar conclusions [52].

## Supporting Information

Figure S1 **GC disparity mapped the terminus region of the** ***S. elongatus*** **chromosome.** Due to its highly recombinant nature, the genome of *S. elongatus* gives rise to a plot that does not display a clear V-shaped curve typical of organisms such as *E. coli*. We reasoned that the terminus region would be present within the vicinity of highest peak of GC disparity (green line) so we looked for a region amenable for integration and inserted a 240 repeat *lacO*-array in a region located at 1.59 Mb in the chromosome.
(TIF)

## References

[1] Lau IF, Filipe SR, Søballe B, Økstad O-A, Barre F-X, et al.. (2003) Spatial and temporal organization of replicating Escherichia coli chromosomes. Molecular microbiology 49: 731–743. Available: http://www.ncbi.nlm.nih.gov/pubmed/12864855. Accessed 2012 Jul 13. 10.1046/j.1365-2958.2003.03640.x 12864855

[2] Berlatzky IA, Rouvinski A, Ben-Yehuda S (2008) Spatial organization of a replicating bacterial chromosome. Proceedings of the National Academy of Sciences of the United States of America 105: 14136–14140. Available: http://www.pubmedcentral.nih.gov/articlerender.fcgi?artid=2544591&tool=pmcentrez&rendertype=abstract. Accessed 2012 Jul 13. 10.1073/pnas.0804982105 PMC2544591 18779567

[3] Toro E, Shapiro L (2010) Bacterial chromosome organization and segregation. Cold Spring Harbor perspectives in biology 2: a000349. Available: http://cshperspectives.cshlp.org/content/2/2/a000349.full. Accessed 2012 Jul 16. 10.1101/cshperspect.a000349 PMC2828278 20182613

[4] Graumann PL, Dame RT, Dorman CJ (2010) The Chromosome Segregation Machinery in Bacteria. Bacterial Chromatin (Google eBook). Springer. 31–48. Available: http://books.google.com/books?id=Esi4D4NjvskC&pgis=1. Accessed 2012 Jul 13.

[5] Haeusser DP, Levin PA (2008) The great divide: coordinating cell cycle events during bacterial growth and division. Current opinion in microbiology 11: 94–99. Available: http://www.pubmedcentral.nih.gov/articlerender.fcgi?artid=2397022&tool=pmcentrez&rendertype=abstract. Accessed 2012 Jul 13. 10.1016/j.mib.2008.02.008 PMC2397022 18396093

[6] Viollier PH, Thanbichler M, McGrath PT, West L, Meewan M, et al.. (2004) Rapid and sequential movement of individual chromosomal loci to specific subcellular locations during bacterial DNA replication. Proceedings of the National Academy of Sciences of the United States of America 101: 9257–9262. Available: http://www.pnas.org/cgi/content/abstract/101/25/9257. Accessed 2012 Jul 16. 10.1073/pnas.0402606101 PMC438963 15178755

[7] Griese M, Lange C, Soppa J (2011) Ploidy in cyanobacteria. FEMS Microbiology Letters 323: 124–131. Available: http://www.ncbi.nlm.nih.gov/pubmed/22092711. Accessed 2012 Nov 15. 10.1111/j.1574-6968.2011.02368.x 22092711

[8] Binder BJ, Chisholm SW (1990) Relationship between DNA cycle and growth rate in Synechococcus sp. strain PCC 6301. J Bacteriol 172: 2313–2319. Available: http://jb.asm.org/cgi/content/abstract/172/5/2313. Accessed 2012 Jul 15. 10.1128/jb.172.5.2313-2319.1990 PMC208864 2110139

[9] Mott ML, Berger JM (2007) DNA replication initiation: mechanisms and regulation in bacteria. Nature reviews Microbiology 5: 343–354. Available: http://dx.doi.org/10.1038/nrmicro1640. Accessed 2012 Jul 16. 10.1038/nrmicro1640 17435790

[10] Løbner-Olesen A, Skarstad K, Hansen FG, von Meyenburg K, Boye E (1989) The DnaA protein determines the initiation mass of Escherichia coli K-12. Cell 57: 881–889. Available: http://dx.doi.org/10.1016/0092-8674(89)90802-7. Accessed 2012 Jul 16. 10.1016/0092-8674(89)90802-7 2541928

[11] Reyes-Lamothe R, Possoz C, Danilova O, Sherratt DJ (2008) Independent positioning and action of Escherichia coli replisomes in live cells. Cell 133: 90–102. Available: http://www.pubmedcentral.nih.gov/articlerender.fcgi?artid=2288635&tool=pmcentrez&rendertype=abstract. Accessed 2012 Jul 13. 10.1016/j.cell.2008.01.044 PMC2288635 18394992

[12] Jensen RB (2006) Coordination between chromosome replication, segregation, and cell division in Caulobacter crescentus. Journal of bacteriology 188: 2244–2253. Available:http://www.pubmedcentral.nih.gov/articlerender.fcgi?artid=1428140&tool=pmcentrez&rendertype=abstract. Accessed 2012 Jul 13. 10.1128/JB.188.6.2244-2253.2006 PMC1428140 16513754

[13] Ebersbach G, Gerdes K (2005) Plasmid segregation mechanisms. Annual review of genetics 39: 453–479. Available: http://www.annualreviews.org.ezp-prod1.hul.harvard.edu/doi/full/10.1146/annurev.genet.38.072902.091252. Accessed 2012 Jul 15. 10.1146/annurev.genet.38.072902.091252 16285868

[14] Allen MM, Stanier RY (1968) Growth and Division of Some Unicellular Blue-green Algae. Journal of General Microbiology 51: 199–202. Available: http://mic.sgmjournals.org/cgi/content/abstract/51/2/199. Accessed 2012 Aug 12. 10.1099/00221287-51-2-199 5652095

[15] Clerico EM, Ditty JL, Golden SS (2007) Specialized techniques for site-directed mutagenesis in cyanobacteria. Methods in molecular biology (Clifton, NJ) 362: 155–171. Available: http://www.ncbi.nlm.nih.gov/pubmed/17417008. Accessed 2012 Jul 13. 10.1007/978-1-59745-257-1_11 17417008

[16] Phillips I, Silver P (2006) A New Biobrick Assembly Strategy Designed for Facile Protein Engineering. Available: http://dspace.mit.edu/handle/1721.1/32535. Accessed 2012 Aug 12.

[17] Lau IF, Filipe SR, Søballe B, Økstad O-A, Barre F-X, et al.. (2004) Spatial and temporal organization of replicating Escherichia coli chromosomes. Molecular Microbiology 49: 731–743. Available: http://doi.wiley.com/10.1046/j.1365-2958.2003.03640.x. Accessed 2012 Jul 15. 10.1046/j.1365-2958.2003.03640.x 12864855

[18] Mackey SR, Ditty JL, Clerico EM, Golden SS (2007) Detection of rhythmic bioluminescence from luciferase reporters in cyanobacteria. Methods in molecular biology (Clifton, NJ) 362: 115–129. Available: http://www.ncbi.nlm.nih.gov/pubmed/17417005. Accessed 2012 Aug 12. 10.1007/978-1-59745-257-1_8 17417005

[19] Viollier PH, Thanbichler M, McGrath PT, West L, Meewan M, et al.. (2004) Rapid and sequential movement of individual chromosomal loci to specific subcellular locations during bacterial DNA replication. Proceedings of the National Academy of Sciences of the United States of America 101: 9257–9262. Available: http://www.pubmedcentral.nih.gov/articlerender.fcgi?artid=438963&tool=pmcentrez&rendertype=abstract. Accessed 2012 Jul 16. 10.1073/pnas.0402606101 PMC438963 15178755

[20] Stuurman N, Amdodaj N, Vale R (2007) Micro-Manager: Open Source software for light microscope imaging. Microscopy Today 15: 42–43. Available: http://www.citeulike.org/group/8437/article/3968410. Accessed 2012 Aug 12.

[21] Image processing with ImageJ (2004). Biophotonics international 11: 36–42. Available:http://igitur-archive.library.uu.nl/med/2011-0512-200507/UUindex.html. Accessed 12 August 2012.

[22] Sliusarenko O, Heinritz J, Emonet T, Jacobs-Wagner C (2011) High-throughput, subpixel precision analysis of bacterial morphogenesis and intracellular spatio-temporal dynamics. Molecular microbiology 80: 612–627. Available: http://www.pubmedcentral.nih.gov/articlerender.fcgi?artid=3090749&tool=pmcentrez&rendertype=abstract. Accessed 2012 Jul 15. 10.1111/j.1365-2958.2011.07579.x PMC3090749 21414037

[23] Hu B, Yang G, Zhao W, Zhang Y, Zhao J (2007) MreB is important for cell shape but not for chromosome segregation of the filamentous cyanobacterium Anabaena sp. PCC 7120. Molecular microbiology 63: 1640–1652. Available: http://www.ncbi.nlm.nih.gov/pubmed/17367385. Accessed 2012 Jul 13. 10.1111/j.1365-2958.2007.05618.x 17367385

[24] Schneider D, Fuhrmann E, Scholz I, Hess WR, Graumann PL (2007) Fluorescence staining of live cyanobacterial cells suggest non-stringent chromosome segregation and absence of a connection between cytoplasmic and thylakoid membranes. BMC cell biology 8: 39. Available: http://www.biomedcentral.com/1471-2121/8/39. Accessed 12012 Jul 13. 10.1186/1471-2121-8-39 PMC2040150 17767716

[25] Gitai Z, Thanbichler M, Shapiro L (2005) The choreographed dynamics of bacterial chromosomes. Trends in microbiology 13: 221–228. Available: http://www.ncbi.nlm.nih.gov/pubmed/15866039. Accessed 2012 Jul 13. 10.1016/j.tim.2005.03.006 15866039

[26] Li Y, Sergueev K, Austin S (2002) The segregation of the Escherichia coli origin and terminus of replication. Molecular microbiology 46: 985–996. Available: http://www.ncbi.nlm.nih.gov/pubmed/12421305. Accessed 2012 Jul 13. 10.1046/j.1365-2958.2002.03234.x 12421305

[27] Pédelacq J-D, Cabantous S, Tran T, Terwilliger TC, Waldo GS (2006) Engineering and characterization of a superfolder green fluorescent protein. Nature biotechnology 24: 79–88. Available: http://www.ncbi.nlm.nih.gov/pubmed/16369541. Accessed 2012 Jul 13. 10.1038/nbt1172 16369541

[28] Gao F, Zhang C-T (2008) Ori-Finder: a web-based system for finding oriCs in unannotated bacterial genomes. BMC bioinformatics 9: 79. Available: http://www.biomedcentral.com/1471-2105/9/79. Accessed 2012 Aug 6. 10.1186/1471-2105-9-79 PMC2275245 18237442

[29] Watanabe S, Ohbayashi R, Shiwa Y, Noda A, Kanesaki Y, et al.. (2012) Light-dependent and asynchronous replication of cyanobacterial multi-copy chromosomes. Molecular Microbiology 83: 856–865. Available: http://doi.wiley.com/10.1111/j.1365-2958.2012.07971.x. Accessed 2012 Jul 16. 10.1111/j.1365-2958.2012.07971.x 22403820

[30] Chang Y-F, Chang C-H (2011) CAGO: a software tool for dynamic visual comparison and correlation measurement of genome organization. PloS one 6: e27080. Available: http://dx.plos.org/10.1371/journal.pone.0027080. Accessed 2012 Aug 11. 10.1371/journal.pone.0027080 PMC3219657 22114666

[31] Webb CD, Teleman A, Gordon S, Straight A, Belmont A, et al.. (1997) Bipolar Localization of the Replication Origin Regions of Chromosomes in Vegetative and Sporulating Cells of B. subtilis. Cell 88: 667–674. Available: http://dx.doi.org/10.1016/S0092-8674(00)81909-1. Accessed 2012 Aug 6. 10.1016/s0092-8674(00)81909-1 9054506

[32] Gordon GS, Shivers RP, Wright A (2002) Polar localization of the Escherichia coli oriC region is independent of the site of replication initiation. Molecular microbiology 44: 501–507. Available: http://www.ncbi.nlm.nih.gov/pubmed/11972786. Accessed 2012 Aug 6. 10.1046/j.1365-2958.2002.02901.x 11972786

[33] Niki H, Hiraga S (1998) Polar localization of the replication origin and terminus in Escherichia coli nucleoids during chromosome partitioning. Genes & development 12: 1036–1045. Available: http://www.pubmedcentral.nih.gov/articlerender.fcgi?artid=316681&tool=pmcentrez&rendertype=abstract. Accessed 2012 Aug 6. 10.1101/gad.12.7.1036 PMC316681 9531540

[34] Mori T, Binder B, Johnson CH (1996) Circadian gating of cell division in cyanobacteria growing with average doubling times of less than 24 hours. Proceedings of the National Academy of Sciences of the United States of America 93: 10183–10188. Available: http://www.pubmedcentral.nih.gov/articlerender.fcgi?artid=38358&tool=pmcentrez&rendertype=abstract. Accessed 2012 Aug 6. 10.1073/pnas.93.19.10183 PMC38358 8816773

[35] Skarstad K, von Meyenburg K, Hansen FG, Boye E (1988) Coordination of chromosome replication initiation in Escherichia coli: effects of different dnaA alleles. J Bacteriol 170: 852–858. Available: http://jb.asm.org/cgi/content/abstract/170/2/852. Accessed 2012 Jul 13. 10.1128/jb.170.2.852-858.1988 PMC210732 2828328

[36] Ha T, Kozlov AG, Lohman TM (2012) Single-Molecule Views of Protein Movement on Single-Stranded DNA. Available: http://www.annualreviews.org.ezp-prod1.hul.harvard.edu/doi/abs/10.1146/annurev-biophys-042910-155351. Accessed 2012 Aug 11. 10.1146/annurev-biophys-042910-155351 PMC3719979 22404684

[37] Bates D (2008) The bacterial replisome: back on track? Molecular microbiology 69: 1341–1348. Available: http://www.pubmedcentral.nih.gov/articlerender.fcgi?artid=2972702&tool=pmcentrez&rendertype=abstract. Accessed 2012 Jul 27. 10.1111/j.1365-2958.2008.06378.x PMC2972702 18673457

[38] Berlatzky IA, Rouvinski A, Ben-Yehuda S (2008) Spatial organization of a replicating bacterial chromosome. Proceedings of the National Academy of Sciences of the United States of America 105: 14136–14140. Available: http://www.pnas.org/cgi/content/abstract/105/37/14136. Accessed 2012 May 9. 10.1073/pnas.0804982105 PMC2544591 18779567

[39] Niki H, Yamaichi Y, Hiraga S (2000) Dynamic organization of chromosomal DNA in Escherichia coli. Genes & Dev 14: 212–223. Available: http://genesdev.cshlp.org/cgi/content/abstract/14/2/212. Accessed 2012 Aug 12. PMC316355 10652275

[40] Jensen RB, Wang SC, Shapiro L (2001) A moving DNA replication factory in Caulobacter crescentus. The EMBO journal 20: 4952–4963. Available: http://dx.doi.org/10.1093/emboj/20.17.4952. Accessed 2012 Jul 16. 10.1093/emboj/20.17.4952 PMC125615 11532959

[41] Reyes-Lamothe R, Wang X, Sherratt D (2008) Escherichia coli and its chromosome. Trends in microbiology 16: 238–245. Available: http://www.ncbi.nlm.nih.gov/pubmed/18406139. Accessed 2012 Jul 13. 10.1016/j.tim.2008.02.003 18406139

[42] Savage DF, Afonso B, Chen AH, Silver PA (2010) Spatially ordered dynamics of the bacterial carbon fixation machinery. Science (New York, NY) 327: 1258–1261. Available: http://www.sciencemag.org/content/327/5970/1258.abstract. Accessed 2012 Mar 6. 10.1126/science.1186090 20203050

[43] Losick R, Shapiro L (1999) Changing Views on the Nature of the Bacterial Cell: from Biochemistry to Cytology. J Bacteriol 181: 4143–4145. Available: http://jb.asm.org. Accessed 2012 May 9. 10.1128/jb.181.14.4143-4145.1999 PMC93912 10400568

[44] Noirot-Gros M-F, Dervyn E, Wu LJ, Mervelet P, Errington J, et al.. (2002) An expanded view of bacterial DNA replication. Proceedings of the National Academy of Sciences of the United States of America 99: 8342–8347. Available: http://www.pubmedcentral.nih.gov/articlerender.fcgi?artid=123069&tool=pmcentrez&rendertype=abstract. Accessed 2012 Jul 13. 10.1073/pnas.122040799 PMC123069 12060778

[45] Sherratt DJ (2003) Bacterial chromosome dynamics. Science (New York, NY) 301: 780–785. Available: http://www.sciencemag.org/content/301/5634/780.full. Accessed 2011 June 23. 10.1126/science.1084780 12907786

[46] Jensen RB, Shapiro L (1999) The Caulobacter crescentus smc gene is required for cell cycle progression and chromosome segregation. Proceedings of the National Academy of Sciences 96: 10661–10666. Available: http://www.pnas.org/cgi/content/abstract/96/19/10661. Accessed 2012 Aug 12. 10.1073/pnas.96.19.10661 PMC17939 10485882

[47] Fogel MA, Waldor MK (2005) Distinct segregation dynamics of the two Vibrio cholerae chromosomes. Molecular microbiology 55: 125–136. Available: http://www.ncbi.nlm.nih.gov/pubmed/15612922. Accessed 2012 Aug 12. 10.1111/j.1365-2958.2004.04379.x 15612922

[48] Sherratt DJ (2003) Bacterial chromosome dynamics. Science (New York, NY) 301: 780–785. Available: http://www.sciencemag.org/content/301/5634/780.abstract. Accessed 2012 Jul 13. 10.1126/science.1084780 12907786

[49] Skarstad K, Boye E, Steen HB (1986) Timing of initiation of chromosome replication in individual Escherichia coli cells. The EMBO journal 5: 1711–1717. Available: http://www.pubmedcentral.nih.gov/articlerender.fcgi?artid=1166998&tool=pmcentrez&rendertype=abstract. Accessed 2012 Jul 13. 10.1002/j.1460-2075.1986.tb04415.x PMC1166998 3527695

[50] Ringgaard S, van Zon J, Howard M, Gerdes K (2009) Movement and equipositioning of plasmids by ParA filament disassembly. Proceedings of the National Academy of Sciences of the United States of America 106: 19369–19374. Available: http://www.pubmedcentral.nih.gov/articlerender.fcgi?artid=2775997&tool=pmcentrez&rendertype=abstract. Accessed 2012 Jul 13. 10.1073/pnas.0908347106 PMC2775997 19906997

[51] Gerdes K, Howard M, Szardenings F (2010) Pushing and pulling in prokaryotic DNA segregation. Cell 141: 927–942. Available: http://www.ncbi.nlm.nih.gov/pubmed/20550930. Accessed 2012 Jul 13. 10.1016/j.cell.2010.05.033 20550930

[52] Jain IH, Vijayan V, O’Shea EK (2012) Spatial ordering of chromosomes enhances the fidelity of chromosome partitioning in cyanobacteria. Proceedings of the National Academy of Sciences of the United States of America: 1211144109–. Available: http://www.pnas.org/cgi/content/abstract/1211144109v1. Accessed 2012 Aug 10. 10.1073/pnas.1211144109 PMC3427121 22869746

