# Supplementary figures and images for: Spatial and Temporal Organization of Chromosome Duplication and Segregation in the Cyanobacterium Synechococcus elongatus PCC 7942
Source: PLoS One. 2012 Oct 24;7(10):e47837. doi: 10.1371/journal.pone.0047837 (PMC3480399; doi:10.1371/journal.pone.0047837)

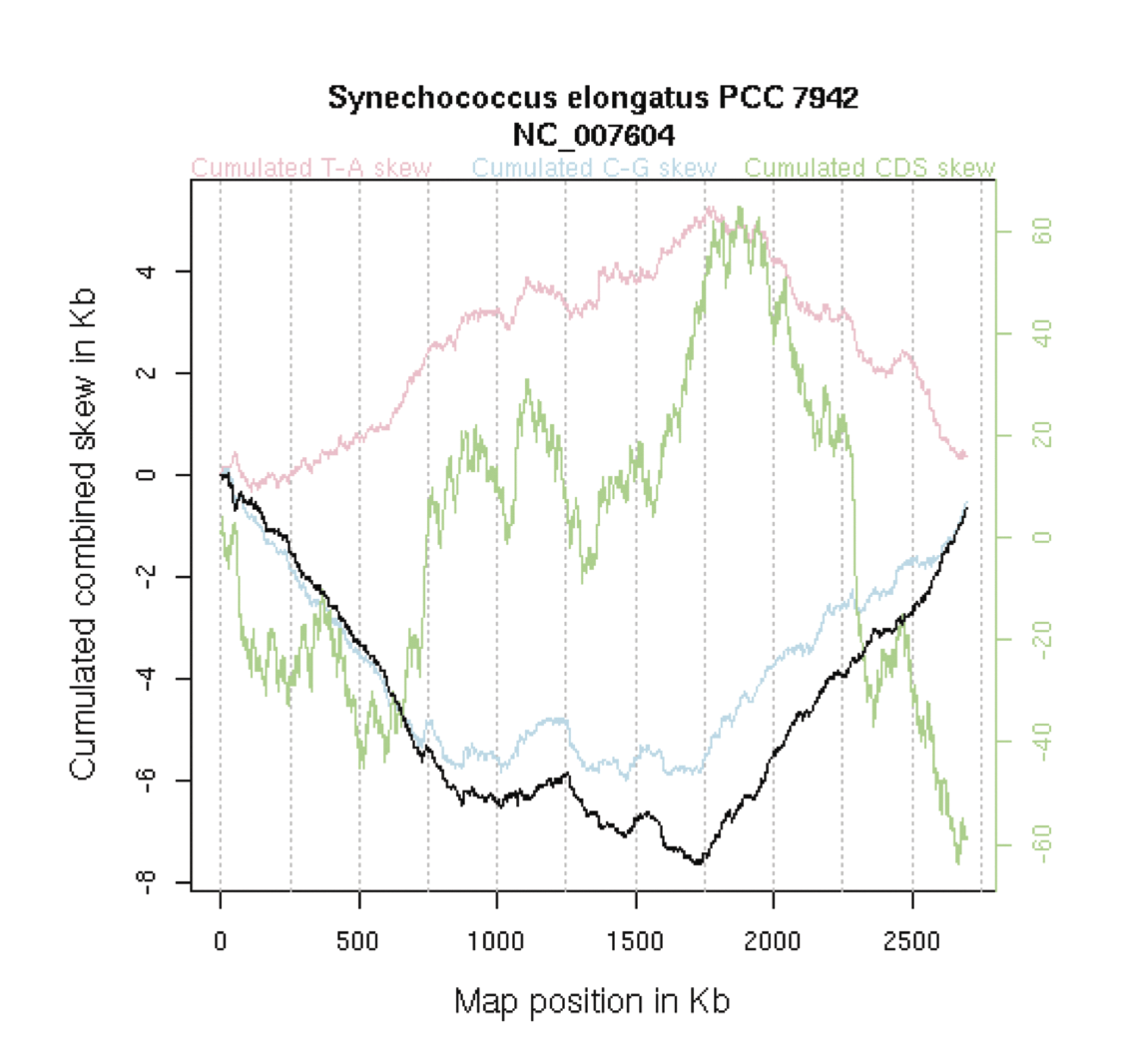

Supplement: Figure S1 — GC disparity mapped the terminus region of the S. elongatus chromosome. Due to its highly recombinant nature, the genome of S. elongatus gives rise to a plot that does not display a clear V-shaped curve typical of organisms such as E. coli. We reasoned that the terminus region would be present within the vicinity of highest peak of GC disparity (green line) so we looked for a region amenable for integration and inserted a 240 repeat lacO-array in a region located at 1.59 Mb in the chromosome. (TIF) [file pone.0047837.s001.tif]
